# Effect of Shot Peening on Redistribution of Residual Stress Field in Friction Stir Welding of 2219 Aluminum Alloy

**DOI:** 10.3390/ma13143169

**Published:** 2020-07-16

**Authors:** Lin Nie, Yunxin Wu, Hai Gong, Dan Chen, Xudong Guo

**Affiliations:** 1 School of Mechanical and Electronic Engineering, Central South University, Changsha 410083, China; nielin@csu.edu.cn (L.N.); gonghai@csu.edu.cn (H.G.); cd1023247758@csu.edu.cn (D.C.); 2 State Key Laboratory of High-Performance Complex Manufacturing, Central South University, Changsha 410083, China; 3 Light Alloy Research Institute, Central South University, Changsha 410083, China; guoxudong@csu.edu.cn

**Keywords:** residual stress, shot peening, friction stir welding, 2219 aluminum alloy

## Abstract

Welding is one of the essential stages in the manufacturing process of mechanical structures. Friction stir welding structure of aluminum alloy has been used as a primary supporting member in aerospace equipment. However, friction stir welding inevitably generates residual stress that promotes the initiation and propagation of cracks, threatening the performance of the welded structure. Shot peening can effectively change the distribution of residual stress and improve the fatigue properties of materials. In this paper, friction stir welding and shot peening are performed on 2219 aluminum alloy plates. The residual stress fields induced by friction stir welding and shot peening are measured by using the X-ray diffraction method and incremental center hole drilling method, and the distribution characteristics of residual stress fields are analyzed. The effect of the pellet diameters and pellet materials used in shot peening on the redistribution of welding residual stress field are investigated. The pellet diameter used in the experiment is in the range of 0.6–1.2 mm, and the pellet material includes glass, steel, and corundum. This study provides guidance for the application of shot peening in friction stir welding structure of 2219 aluminum alloy.

## 1. Introduction

The application of high strength aluminum alloy can effectively reduce the structural weight of spacecraft. Basically, 2219 aluminum alloy has been selected as the main structural material for the fuel and oxidant tank of the new-generation launch vehicle due to its excellent high/low-temperature mechanical properties [1]. However, the tank is difficult to be manufactured at one time because of its complex structures, which requires the use of welding technology to complete the assembly and connection. In fact, the longitudinal seams of the cylinder parts are connected by friction stir welding.

Friction stir welding, as a new solid-state joining technology, has been widely used in the aerospace manufacturing [2]. In the welding process, the tool pin is inserted into the joint of the workpiece, and then the high-speed friction between the tool and the workpiece generates heat, which softens the material and complete the welding. Although the main problem in welded structures is that the harmful residual stresses will inevitably be generated during the welding process [3,4,5]. Lemos et al. [6] performed friction stir welding with different process parameters to analyze the characteristics of residual stresses and observed higher difference of the residual stresses state between the advancing side and the retreating side. Huang et al. [1] measured the residual stresses in variable polarity plasma arc welded joint and friction stir welded joint on propellant tank of aluminum alloy by using the indentation strain-gauge method. The results showed that there are large residual tensile stresses in the center and inner areas of the friction stir welded joint, and the weak spot of the tank appears in the region of maximum tensile stress. Yu et al. [7] developed an analysis model for friction stir welding to obtain the distribution of residual stresses. The results indicated that the tensile stresses are concentrated in the welded zone.

Welding residual stresses promote the initiation and failure of fatigue cracks and even cause instability and fracture of components in complex service environments [8,9,10,11], which greatly affects the performance of components. Ferro et al. [12] observed that the fatigue strength of welded joints is sensitive to the initial residual stresses caused by welding under high cyclic load. Lee et al. [13] established a fatigue damage model to calculate the fatigue crack growth of welded joints. Compared with the test results, it was found that the welding residual stresses cannot be ignored in the estimation of the fatigue life of the weld. Fratini et al. [14] investigated the influence of the residual stresses on the fatigue crack propagation in welded joints of 2024 aluminum alloy and found that the fatigue crack propagation behavior is related to the welding residual stresses.

Welding is the only option for joining different parts in some industrial applications [15], but the residual stresses profile in the friction stir welded joints affects the quality of the joints. Therefore, it is of practical significance to study the real state of welding residual stresses for eliminating the residual stresses and evaluating the service performance of welded structure.

To improve the fatigue life of industrial parts and components, various surface treatment technologies have been developed in recent decades [16,17,18,19]. Shot peening is a well-known surface strengthening technology, which can significantly enhance the fatigue strength and effectively prevent the failure of components. This method is flexible and can be applied to almost any shape of components and has been widely used in industry [20,21]. The application of shot peening has great attraction to the welded joints, because of the low fatigue resistance of welded joints due to the high stress concentration.

The mechanism of shot peening is the process of impacting the material surface with high-speed pellets to plastically deform the impacted surface. During the process, residual compressive stresses beneficial to improve the fatigue performance are introduced into the components [22,23]. Therefore, for improving the shot peening technology and component performance, it is of great significant to analyze the relationship between the shot peening process and the residual stress field in the components and grasp the distribution of the residual stress field introduced by shot peening. Chen et al. [24] shot peened the surface of 2507 stainless steel, and the measurement results indicated that high residual compressive stresses are induced on the surface layer after shot peening, which is helpful to improve the fatigue performance of components. Wu et al. [25] studied the effect of shot peening parameters on the residual stress of aluminum alloy. Zhang et al. [26] presented shot peening experiments with different process parameters and established a finite element model to study the residual stress distribution. The results indicated that the residual compressive stresses are proportional to the shot peening speed, and the compressive stress depth increases with the increase of coverage. Zhou et al. [27] proposed a numerical method to analyze the residual stresses introduced by shot peening, and the experiment verified the validity of the model. Liu et al. [28] used SPH method to simulate shot peening to eliminate welding residual stresses. The evolution of residual stresses was proposed, and the change of welding residual stresses before and after shot peening was analyzed. In addition, the effect of shot peening velocity on residual stresses is investigated.

Although many scholars have studied a lot of shot peening [29,30,31], the experimental research on shot peening of aluminum alloy-welded structures is rarely involved. In addition, operators mainly depend on experience to select shot peening parameters in the manufacturing practice [32]. There is a lack of experimental basic research on the variation of residual stress field under various shot peening parameters.

In this paper, the experiments of friction stir welding and shot peening on 2219 aluminum alloy plates are carried out at the State Key Laboratory of High-Performance Complex Manufacturing, Central South University. The distribution characteristics of residual stress field under different process parameters are measured, and the effect of shot peening on the redistribution of residual stress field is analyzed, which provide experimental data for determining the optimal shot peening process parameters.

## 2. Materials and Methods

### 2.1. Materials

The 2219 aluminum alloy used in the experiment is high-strength aluminum alloy that belongs to Al-Cu-Mn series, which can be enhanced by heat treatment. However, small precipitates of Cu at high temperature may be the cause of cracking and the limitation of weldability. The chemical composition of 2219 aluminum alloy as listed in Table 1. The yield strength at room temperature was 352 MPa, the tensile strength was 455 MPa, and the elongation was 10% [33].

### 2.2. Friction Stir Welding

**Figure 1:**
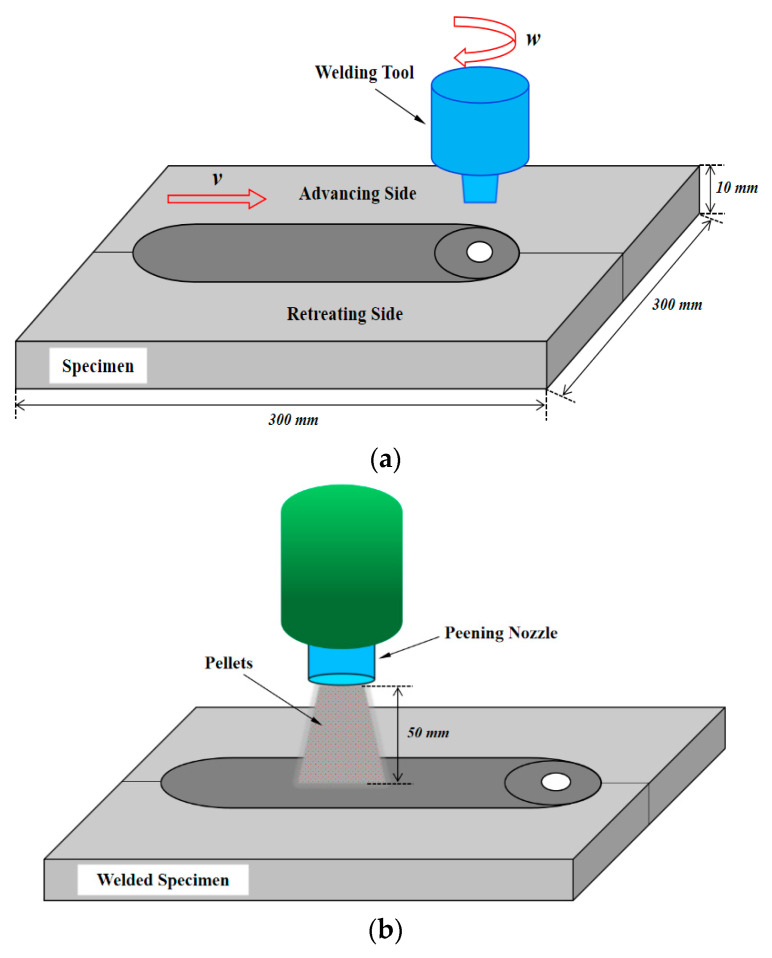
Diagram of friction stir welding and shot peening: (**a**) friction stir welding process and (**b**) shot peening process.

Two 2219 aluminum alloy plates with dimensions of 300 mm × 150 mm × 10 mm were used for friction stir welding experiments, as shown in Figure 1. The dimensions of the tool used for welding were 15 mm in radius of shoulder, 9 mm in radius of pin, and 9.7 mm in length of pin. The welding parameters were 800 rpm in rotating speed and 100 mm/min in welding speed. This group of parameters was selected because the welding parameters need to be matched with the tool to ensure the welding quality. However, our laboratory has carried out relevant tests before and found that using these parameters can obtain better welding quality. Although this may not be the best, there were no surface and internal defects in the weldment. The welding experiments were performed on the friction stir welding equipment developed by Central South University.

### 2.3. Shot Peening

**Figure 2:**
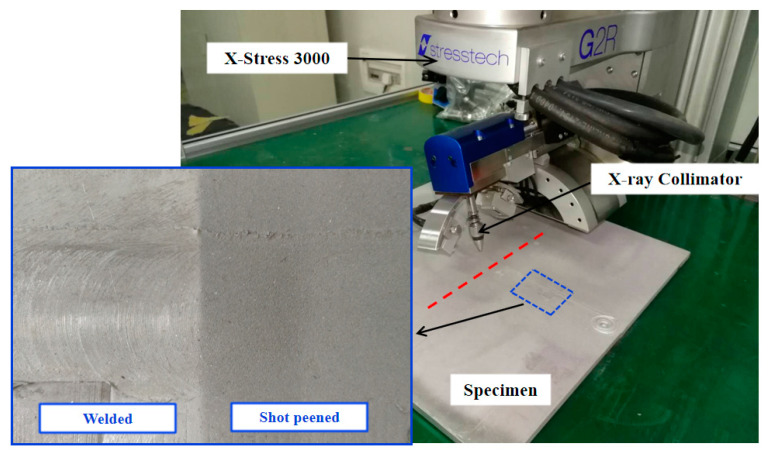
The process of measuring residual stress by XRD.

After the welding, the shot peening was performed on the surface of the welded specimens, as shown in Figure 1. The shot peening machine used in the experiment was CJ-106P sand-blasting machine produced by Dongguan Changjie Automation Equipment Co., Ltd., Dongguan, China. Its working pressure was 0.3–0.8 MPa and the nozzle diameter was 12 mm. In the shot peening experiments, a shot pressure of 0.4 MPa and a shot distance of 50 mm were used uniformly. The shot strategy was perpendicular to the surface of the welded specimens, and the parts that were not shot-peened were protected while shot peening. The pellets with different materials and different diameters were selected to perform shot peening on the surface of the welded specimens. The physical parameters of pellets and experimental parameters of shot peening are listed in Table 2 and Table 3. The chemical compositions (wt.%) of the steel shot were: C 0.07, Si 0.8, Mn 1.2, P 0.045, S 0.03, Cr 18, Ni 10, and Fe balance. The surface after shot peening is shown in Figure 2.

### 2.4. Residual Stress Measurement

The residual stresses on the surface and inside of the welded specimens before and after shot peening were measured by the method of X-ray diffraction (XRD) and incremental center hole drilling (ICHD), respectively, to investigate the effect of shot peening on the redistribution of welding residual stresses.

The residual stresses on the surface of the specimens were measured by Proto X-ray diffraction stress analyzer (X-Stress 3000) equipped with a collimator of 2 mm diameter. The measurement positions are shown as the red dotted line in Figure 2. The residual stresses measurements were performed by using the traditional d-spacing versus $\sin^{2}\theta$ technique [4], which is based on the elliptical regression of $d-\sin^{2}\theta$ plot to calculate the error bars for each X-ray residual stress measurement. The diffractometer parameters are listed in Table 4. Alignment of the X-ray diffractometer was confirmed using a stress-free aluminum powder calibration sample from Proto Manufacturing prior to measuring residual stresses on the plate.

**Figure 3:**
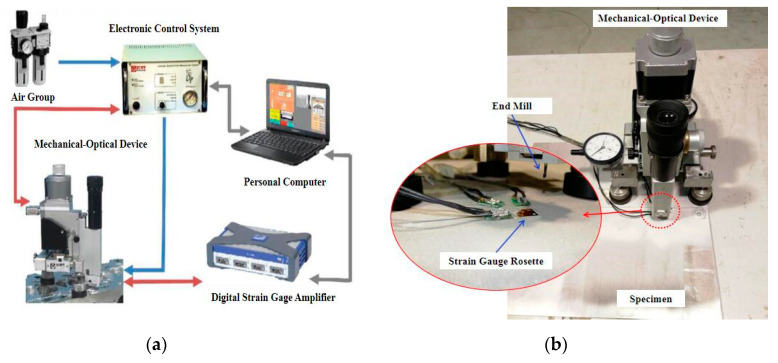
The schematic diagram of measuring residual stress by incremental center hole drilling (ICHD): (**a**) automatic hole drilling system and (**b**) measurement of residual stress.

Shot peening affected not only the surface residual stresses of the specimens but also the internal residual stresses in the depth of the specimens. Therefore, data on residual stresses were also collected as a function of depth by using ICHD method. The automatic hole drilling system MTS3000 manufactured by SINT Technology company was used to measure the internal residual stresses. It is equipped with an optical calibration system to ensure that the drilled hole is located at the center of the strain gauge rosette. The end mill drilled holes with a depth of 2 mm in 20 steps at a speed of 20,000 rpm and a feed rate of 0.2 mm/min. The strain in the hole with diameter of 1.6 mm was measured between each increment of 0.1 mm to obtain high-quality full field strain data, and then the original residual stress was accurately calculated based on the integration method [15,19]. Figure 3 shows the automatic hole drilling system and the measurement of residual stress.

## 3. Results and Discussion

### 3.1. Distribution of Welding Residual Stress

**Figure 4:**
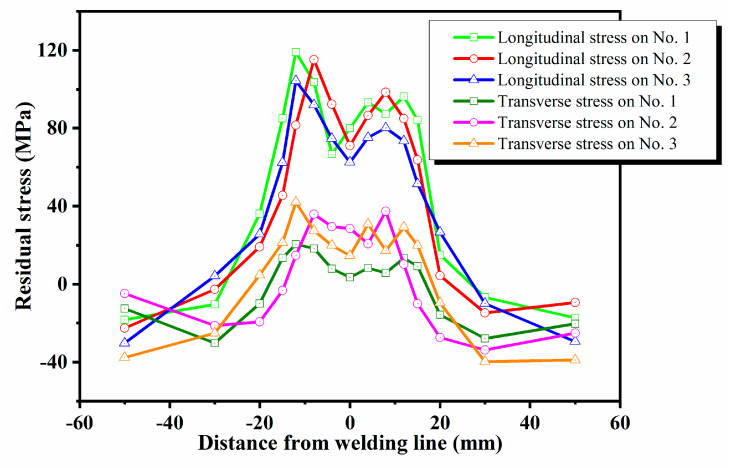
Residual stress distribution on the surface.

Figure 4 shows the residual stress distribution on the surface of the friction stir welded specimens. The same welding parameters were used for the specimens No. 1–No. 3. It can be seen that the residual tensile stresses occur in the weld zone, and its profile is asymmetric with respect to the weld centerline, which is shown in the fact that the residual tensile stresses on the advancing side of the tool shoulder is higher than that on the retreating side. The peak value of tensile stresses on the advancing side is about 20~30% higher than that on the retreating side. The similar trend was observed in the study by Zhan et al. [34]. In this paper, the maximum tensile stress is located near the edge of the shoulder on the advancing side, reaching 119.2 Mpa, which is about 33.5% of the yield strength of the base material. The residual tensile stresses decrease rapidly from the weld zone to the base metal on both sides. Especially in the heat-affected zone with large stress gradient, the residual tensile stress rapidly transforms into residual compressive stress up to the base metal, so as to achieve self-balance of the residual stress field. The longitudinal residual stresses in AA2219-T87 friction stir welded joints were 73–138 MPa reported by Du et al. [35], which are very close to the measurement results in this work.

Compared with the longitudinal residual stresses, the transverse residual stresses were lower than that of the same point on each specimen. As expected, the transverse residual stresses were relatively small, ranging from −39 to 42 MPa. Interestingly, the transverse residual stresses do not show a significantly asymmetrical M-shaped profile. The results showed that the longitudinal residual tensile stresses were the main residual stresses generated by friction stir welding. The work of Brewer et al. [4] and Zhan et al. [34] also shows that the longitudinal residual stresses were much larger than the transverse residual stresses. Therefore, the following discussions will focus on the longitudinal residual stresses.

On the one hand, friction stir welding is a nonuniform heating and cooling process. During the welding process, the thermal expansion of the material in the weld zone generates thermal stresses. The cooling shrinkage of the material in the weld zone during cooling will be restricted by the surrounding materials, resulting in tensile stresses, which will eventually be retained. On the other hand, the shear force of the tool has different effects on the advancing side and the retreating side. During the welding process, the material in the weld zone flows partly toward the advancing side and partly toward the retreating side under the stirring action of the tool. This causes the material on the advancing side to be subjected to tensile stresses and the material on the retreating side to be subjected to compressive stresses. As a result, after the cooling is completed, the residual tensile stresses on the advancing side is greater than that on the retreating side. The above two reasons are the main factors for the asymmetric distribution of welding residual tensile stresses.

**Figure 5:**
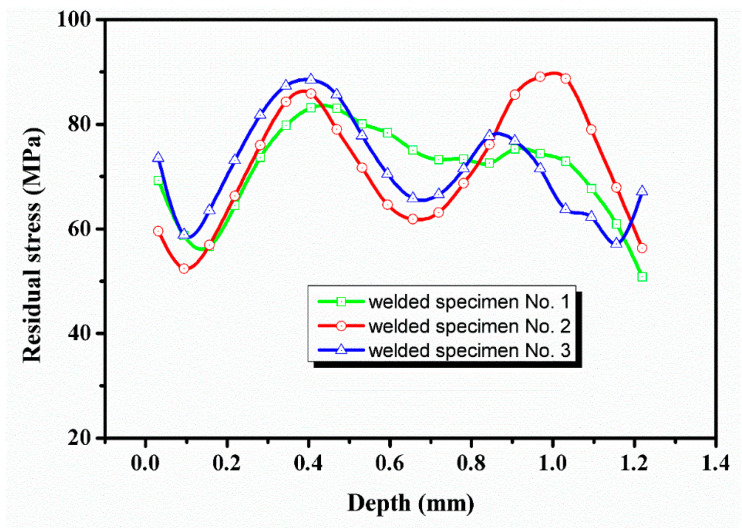
Residual stress distribution along the thickness.

In this paper, the internal residual stresses along the thickness direction in the welded specimens are measured by using ICHD method, as shown in Figure 5. It can be seen that the residual stresses were tensile stresses when inside the weld zone and were evenly distributed between 51 and 89 MPa along the thickness direction.

### 3.2. Effect of Shot Peening on Redistribution of Surface Residual Stress

**Figure 6:**
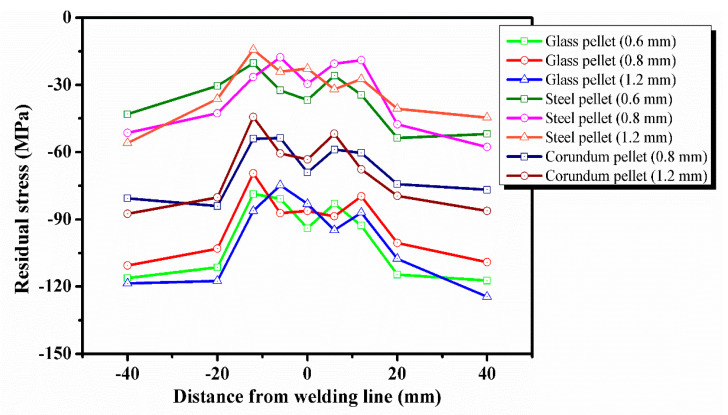
Effect of shot peening on the redistribution of residual stress on the surface.

Figure 6 shows the residual stress redistribution on the surface of the welded + shot peened specimens. In the shot peening experiment, the welded specimen No. 1 was shot peened by glass pellets, the welded specimen No. 2 was shot peened by steel pellets, and the welded specimen No. 3 was shot peened by corundum pellets.

Figure 4 shows that there are large residual tensile stresses in the weld zone of the welded specimens. However, shot peening effectively eliminates the residual tensile stresses and redistributes the residual stresses to achieve balance. It can be observed from Figure 6 that the residual stresses on the surface of the welded specimens after shot peening was in the state of compressive stresses, and its distribution was an asymmetrical M-shaped with respect to the weld centerline. This shows that the compressive stresses introduced by shot peening offsetted the welding tensile stresses, and finally, the compressive stresses were retained. Shot peening effectively reduced the stress gradient in the weld zone and made the residual stress redistributed more uniform. The numerical simulation by Liu et al. [28] also shows that shot peening can eliminate the residual tensile stresses in the weld zone, with the largest reduction being 99.12%.

**Figure 7:**
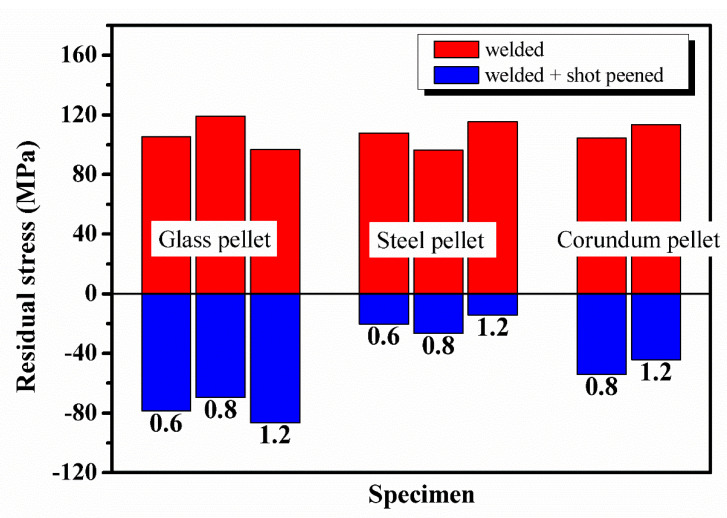
The peak value of welding residual stress is before and after shot peening.

It was observed from Figure 7 that changing the pellet diameter of the same material had no significant effect on the peening effect of the specimen surface. Dieng et al. [21] used three pellet diameters (0.4, 0.8 and 1.2 mm) in the shot peening simulation and found that the compressive stresses on the surface of the specimen were basically constant with only changing the pellet diameter. In this work, the maximum tensile stress in the weld zone was reduced to −78.7, −69.5 and −86.3 MPa, when the glass with pellet diameters of 0.6, 0.8 and 1.2 mm, respectively, were used for shot peening. Although the effect of shot peening to eliminate welding residual tensile stresses was obvious, the residual compressive stresses introduced by shot peening with different pellet diameters did not change much. Shot peening with corundum and steel also showed the same results. This showed that the variation of pellet diameter hardly affected the magnitude of residual compressive stresses introduced by shot peening on the surface of the welded specimens.

However, comparing the shot peening results of three pellet materials with the same diameter, it was observed that the effect of pellet material on the residual compressive stresses on the surface was significant. When the glass, steel, and corundum with a pellet diameter of 1.2 mm were used for shot peening, it was found that the residual compressive stress introduced by shot peening at the position of the peak tensile stress in the weld zone was −182.9, −129.6, −158 MPa, respectively. The comparison of shot peening results showed that the residual compressive stresses introduced by glass on the surface of the welded specimens was the biggest, which was about 1.4 times that of steel and 1.2 times that of corundum.

Shot peening introduced residual compressive stresses on the surface of the welded specimens, but changing the pellet diameter did not affect the magnitude of the introduced residual compressive stresses. The residual compressive stresses introduced by shot peening were significantly affected by the pellet material. Glass could introduce the biggest residual compressive stress on the surface than corundum and steel.

### 3.3. Effect of Shot Peening on Redistribution of Internal Residual Stress

**Figure 8:**
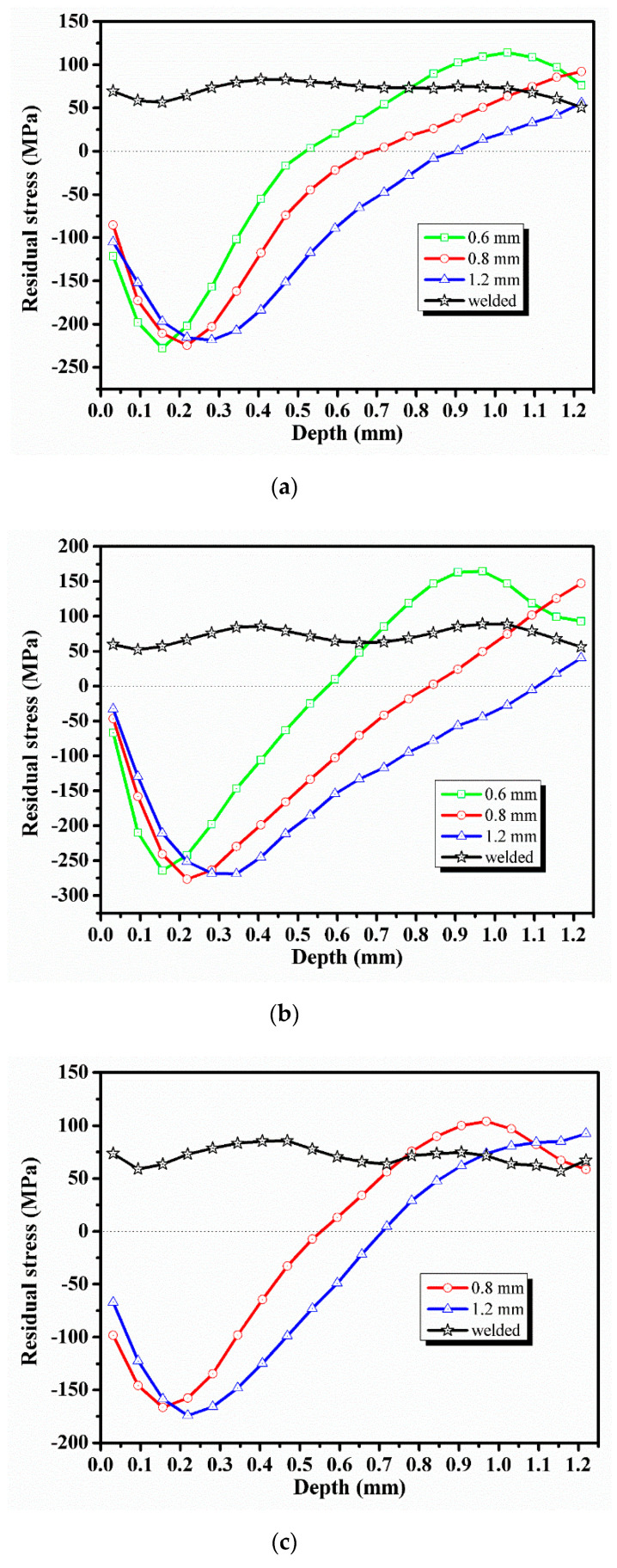
Effect of shot peening on the redistribution of internal residual stress: (**a**) shot peening with glass, (**b**) shot peening with steel, and (**c**) shot peening with corundum.

Figure 8 shows the effect of shot peening on the redistribution of internal residual stresses in the welded specimens. It was observed that shot peening resulted in the redistribution of internal residual tensile stresses. A large amount of residual compressive stresses was introduced within a thickness range of at least 0.5 mm from the specimen surface to form a beneficial residual compressive stress layer. Even after reaching a depth of about 0.7 mm, the residual stress after shot peening was still lower than the welding residual tensile stress. However, the residual compressive stresses introduced by shot peening gradually reduced by moving within the thickness range. After exceeding a certain depth, the residual compressive stress was transformed into residual tensile stress and gradually increased with depth. Finally, the residual stresses after shot peening presented S-shaped distribution along the thickness direction. The similar trend was observed in shot peening simulation of steel, in work by Dieng et al. [21], Zhou et al. [27], and Wu et al. [29]. The literature indicated that the surface compressive stresses were introduced by the elastic deformation, whereas the internal compressive stresses were introduced by the plastic deformation, and their magnitudes were mainly related to the material being shot peened [23].

The internal residual stress field introduced by shot peening is characterized by the maximum residual compressive stress ($\sigma_{m}$), the position of the maximum residual compressive stress (*f*), and the depth of the residual compressive stress layer (*d*). The shot peening results of three pellet materials are listed in Table 5.

The experimental results showed that good peening effect could obtained by using glass, steel, and corundum, and a large amount of residual compressive stresses was introduced inside the welded specimens. By comparing the data in Table 5, it was found that when the glasses with pellet diameters of 0.6, 0.8 and 1.2 mm were used for shot peening, the $\sigma_{m}$ was −232.8, −224.6 and −218.3 MPa; the *f* was 0.158, 0.212 and 0.261 mm; and the *d* was 0.519, 0.687 and 0.9 mm, respectively. These results showed that when a larger pellet was used, the *f* was deeper, and the *d* was also deeper. However, the $\sigma_{m}$ introduced by shot peening had no obvious change with the increase of diameter under the same pellet material. Wu et al. [25] numerically simulated the effect of shot peening on 2024 aluminum alloy and obtained the results consistent with this paper, and they proposed that the residual stress distribution was affected by the pellet diameter, shot peening velocity, and incident angle.

The distribution of residual stress field shown in Figure 8 and the characteristics of the residual stress field shown in Table 5 indicated that changing the pellet material under the same diameter had a significant effect on the $\sigma_{m}$, *f*, and *d*. When the glass, steel, and corundum with a pellet diameter of 1.2 mm were used for shot peening, the $\sigma_{m}$ was −218.33, −268.82 and −174.1 MPa; the *f* was 0.26, 0.315 and 0.228 mm; the *d* was 0.9, 1.11 and 0.708 mm, respectively. These results showed that the biggest $\sigma_{m}$, the deepest *f*, and the deepest *d* can be obtained by shot peening with steel under the same pellet diameter, whereas the peening effect of glass was better than that of corundum.

The peening effect is affected by the kinetic energy of the pellet. According to the equation $E=\frac{1}{2}mv^{2}$ and $m=\frac{4}{3}\pi \rho r^{3}$, it is known that the kinetic energy of the pellet is mainly related to the density and radius, when the velocity of the shot is constant. Therefore, the kinetic energy of the pellet with the same material increases with the increase of its radius. The impact of the pellet with a larger radius on the welded specimen will cause greater plastic deformation and result in deeper residual compressive stress layer. However, as the radius of the pellet increases, the maximum residual compressive stress does not simply increase or decrease linearly. This may be because the maximum residual compressive stress is close to saturation under the condition of high peening strength.

The mass of the pellet determines its kinetic energy. The density of steel is the largest of the three pellets, which means that the kinetic energy of the steel pellet is greater due to its greater mass under the same volume. Therefore, the steel pellet with higher kinetic energy can release more energy when impacting the welded specimen, so that the plastic deformation occurs deeper in the material.

Glass and corundum are brittle materials, which are easy to be broken under the condition of high peening strength, resulting in a part of the impact energy to be loss due to the fragmentation of the pellet. Although the hardness of steel is the smallest of the three pellets, its kinetic energy is converted more into the plastic deformation energy in the material. Therefore, the maximum residual compressive stress introduced by shot peening with steel will be greatest.

In summary, the variation of pellet diameter has no significant effect on the maximum residual compressive stress inside the specimen. However, an increase in the pellet diameter will cause the depth of the residual compressive stress layer introduced by the shot peening to increase significantly, and the position of the maximum residual compressive stress will also shift inward as the pellet diameter increases. Compared with glass and corundum, shot peening with steel introduces the biggest internal residual compressive stress and the deepest residual compressive stress layer.

## 4. Conclusions

In this paper, the experiments of friction stir welding and shot peening were carried out on 2219 aluminum alloy plates, and the distribution of residual stress was measured. The effects of pellet material and pellet diameter on welding residual stress were investigated. The following conclusions are drawn:

(1) Longitudinal residual tensile stresses are the main residual stresses generated by friction stir welding, and its profile is asymmetric with respect to the weld centerline. The peak tensile stress on the advancing side is about 20~30% higher than that on the retreating side. The maximum residual tensile stress is located near the edge of the shoulder on the advancing side, which is about 33.5% of the yield strength of the base metal.
(2) The variation of pellet diameter hardly affects the magnitude of residual compressive stresses introduced by shot peening in the welded specimens, but the position of the maximum residual compressive stress and the depth of the residual compressive stress layer will deepen with the increase of pellet diameter.
(3) Shot peening with glass can introduce the biggest residual compressive stress on the surface of the welded specimens than corundum and steel. Shot peening with steel can introduce the biggest internal residual compressive stress and the deepest residual compressive stress layer, whereas the peening effect of glass is better than that of corundum.
(4) Shot peening techniques can be applied as a simple and practical method to treat the friction stir welded structures of aluminum alloy to obtain favorable benefits.

## Figures and Tables

**Table 1:** Composition of the 2219 aluminum alloy.

Element	Cu	Mn	Fe	Si	Ti	Mg	Zr	Al
wt.%	5.8~6.8	0.2~0.4	0.3	0.2	0.02~0.1	0.02	0.1~0.25	Bal.

**Table 2:** Physical properties of pellet materials.

Pellet Materials	Density (kg/cm^3^)	Elastic Modulus (GPa)	Poisson’s Ratio	Mohs Hardness
Glass	2700	55	0.25	6.5
Steel	7750	206	0.3	5.5
Corundum	3900	–	0.24	10

**Table 3:** Shot peening process parameters.

Welded Specimen	Pellet Material	Pellet Diameter (mm)	Incident Angle	Peening Time (min)
No. 1	Glass	0.6	90°	1
	Glass	0.8	90°	1
	Glass	1.2	90°	1
No. 2	Steel	0.6	90°	1
	Steel	0.8	90°	1
	Steel	1.2	90°	1
No. 3	Corundum	0.8	90°	1
	Corundum	1.2	90°	1

**Table 4:** The X-ray diffractometer parameters.

Diffractometer Parameters	Specification/Values
Tube type	Cr
{hkl} reflection	Al {311}
Bragg angle for diffraction (2θ)	139.3°
Current	6.7 mA
Voltage	30 kV
Exposure time	10 s
Collimator diameter	2 mm
Collimator distance	10.14 mm
Tilt angle	−45° to 45°
Number of tilts	5/5
Rotation angles	0°, 90°

**Table 5:** Characteristics of residual stress fields with different shot peening parameters.

Pellet	Diameter	$\sigma_{m}\;(\mathrm{mm})$	*f* (mm)	*d* (mm)
Glass	0.6	−232.8	0.158	0.519
	0.8	−224.6	0.212	0.687
	1.2	−218.3	0.261	0.9
Steel	0.6	−263.7	0.169	0.576
	0.8	−276.4	0.233	0.836
	1.2	−268.5	0.315	1.11
Corundum	0.8	−166.3	0.172	0.553
	1.2	−174.1	0.228	0.708
